# Feasibility of replacing face‐to‐face with telephone interviews for the World Mental Health Qatar survey during the COVID‐19 pandemic

**DOI:** 10.1002/mpr.2009

**Published:** 2024-05-10

**Authors:** Salma M. Khaled, Iman Amro, Lina Bader, John Lee Holmes, Abdoulaye Diop, Kien Le Trung

**Affiliations:** ^1^ Department of Population Medicine College of Medicine Qatar University Doha Qatar; ^2^ Social and Economic Survey Research Institute Qatar University Doha Qatar

**Keywords:** COVID‐19 pandemic, field cost, response rate, survey mode, world mental health

## Abstract

**Objectives:**

We investigated the feasibility of replacing face‐to‐face with telephone interviews conducted as part of the World Mental Health Qatar (WMHQ) survey and discuss the main methodological changes across the two pilots that were subsequently implemented in the full‐scale WMHQ telephone survey.

**Methods:**

We assessed the net mode effect by comparing the lifetime prevalence estimates of the main mental disorder classes (mood and anxiety disorders) and a number of disorders across the two survey pilots conducted prior to and post‐pandemic.

**Results:**

The main differences in terms of methodology for both pilots stemmed from differences in the survey mode, including questionnaire length, study recruitment method, and fielding team size and structure. These factors influenced response rates and costs. However, the lifetime prevalence estimates and other key indicators of survey results did not differ across modes.

**Conclusions:**

Our findings confirm the comparability of data collected via telephone and face‐to‐face modes, supporting the adoption of telephone surveys for future mental health studies, particularly in the context of pandemics. They also confirm the feasibility of changing or mixing modes depending on field conditions in future psychiatric epidemiological research.

## 1 INTRODUCTION

The World Mental Health (WMH) Survey initiative is a consortium (WHO, n.d.) that developed and applied methodological standards to estimate the prevalence and identify the correlates of mental disorders nationally and globally (Frounfelker et al., 2018; Kessler et al., 2009; Kessler et al., 2009; McGrath et al., 2023). Between 2020 and 2022, the COVID‐19 pandemic significantly disrupted the entire research community across the world. During this period, many researchers were confronted with the difficult decision of whether and how to proceed with their surveys, given that the pandemic presented significant challenges to traditional fieldwork operations like face‐to‐face interviewing. As a result, many official surveys suspended their data collection efforts in response to pandemic‐related physical distancing guidelines. For example, a survey conducted by the United Nations Statistics Division and the World Bank reported that in May 2020, 96% of national statistical offices either partially or completely halted face‐to‐face data collection activities (Inter‐Secretariat Working Group on Household Surveys, 2020).

WMH surveys were no exception to this trend. The COVID‐19 outbreak triggered many changes, including the transition to remote surveying. National statistical offices resorted to remote data collection tools as the primary means for maintaining continuity in survey production (Gourlay et al., 2021). Since most psychiatric epidemiology surveys, including the WMH surveys, relied on face‐to‐face interviews, the change to remote modes of data collection was a significant one. In addition to flexibility, face‐to‐face interviewing is considered the best survey mode in terms of data quality and response rates (Hox & De Leeuw, 1994b; Lavrakas, 2008; Schröder, 2016; Tom W., 1984). Furthermore, this mode of data collection enables interviewers to observe both verbal and nonverbal response cues from respondents and to take direct measurements if needed. It also allows for longer surveys. Due to these advantages, face‐to‐face interviewing is considered “the gold standard” for survey research practice.

Telephone interviews are considered the most effective alternative to face‐to‐face interviews for ensuring continuity of survey research activities if a probability sample is required (Groves, 2009). In the context of the COVID‐19 pandemic, it was hoped that telephone surveys could be applied to carry out WMH surveys, much like telepsychiatry effectively served patients with pre‐existing mental health disorders during the pandemic (Li et al., 2021). Nevertheless, because all WMH surveys used the same standardized procedures for sampling, interviewing, and data analysis (Kessler et al., 2009), concerns emerged regarding whether a shift in survey mode would affect the comparability of results between WMH surveys conducted before and during the pandemic.

To compound these concerns, several published studies have suggested that telephone interviews were ineffective in gathering accurate survey data on sensitive topics (Gross et al., 2018; Gupta & Pathak, 2018; Montemurro & Riehman‐Murphy, 2019; Taylor et al., 2018). Other reported issues included an increase in acquiescence and extremeness in telephone interview responses compared to face‐to‐face surveys (Groves & Kahn, 1979; Jordan et al., 1980). On the contrary, some studies found no differences in compliance, reliability of responses, or outcomes between these two main survey modes (Kennedy et al., 2016, 2017; Marel et al., 2015; Taylor et al., 2016). Historically, during the 1960s and 1970s, multiple studies addressed concerns about the methodological limitations of telephone surveys (Groves & Kahn, 1979; Hochstim, 1967; Sudman & Bradburn, 1974). Over the subsequent years, telephone surveys became more prevalent in psychiatric epidemiology (Stefl, 1984). Finally, there is also some evidence supporting phone surveys as more effective than either mail‐in or face‐to‐face modes in community psychiatric surveys (Conwell et al., 2018; Fenig et al., 1993; Hinkle & King, 1978).

Researchers in Qatar, in consultation with experts from the WMH consortium, recognized the need to change the survey mode for their WMH study in response to the COVID‐19 pandemic. Since the change happened suddenly, it was not possible to conduct experimental comparisons to assess the reliability and validity of the two survey modes. Therefore, the present study aimed to investigate the feasibility of replacing traditional face‐to‐face interviews with phone interviews for Qatar's national mental health survey as part of the WMHS consortium. This involved redesigning and comparing results between the two pilots before subsequently implementing the revised methodology in the full‐scale production of the WMH Qatar (WMHQ) survey. In this paper, we explore the feasibility of adapting data collection methods under conditions of necessity in the context of WMH surveys.

## 2 METHODS

### 2.1 The World Mental Health Qatar (WMHQ)

Face‐to‐face interviews were used for the first pilot survey, which was conducted in early 2020, before the onset of the pandemic. The methodological procedures used in the initial face‐to‐face pilot survey are fully described in a separate published article (Khaled et al., 2021). However, due to the COVID‐19 pandemic in Qatar, telephone was chosen instead of face‐to‐face interviews as the main mode of survey data collection. This revised methodology was tested in a second pilot survey conducted later that same year.

The telephone methodology employed in the second pilot survey served as the basis for the procedures used in the full WMHQ survey. A comprehensive description of these procedures can be found in a separate article published in this issue (Khaled et al., 2024).

Table 1 summarizes the main methodological similarities and differences between the two pilot surveys of the WMHQ study. As shown in Table 1, the two pilot surveys were similar in many design‐related respects, including study target population, questionnaire, and quality control system. The main differences in methodological aspects of both pilots stemmed from differences in the survey mode. These differences in turn influenced other aspects of the study, including questionnaire length, study recruitment method, and the size and structure of the fielding team (Table 1).

**TABLE 1:** Main methodological similarities and differences in the face‐to‐face (pre‐pandemic) and phone (post‐pandemic) pilots conducted as part of the World Mental Health Qatar Survey.

Methodological variables	Face‐to‐face pilot design	Telephone pilot design
Timeframe	Pre‐pandemic: 16 January – February 9, 2020 (total number of 22 days)	Post‐pandemic: October 21, 2020 – December 20, 2020 (total number of 60 days)
Sampling method (frame and sampling design)	Based on a sampling frame of all housing units in Qatar, a stratified sample of households were constructed from eight municipalities	With support from cell phone providers in Qatar, a stratified probability sample was constructed based on list‐assisted dialing
Sample size	345 completed interviews	427 completed interviews
Study population	Representative sample of Arabic speakers only: Qatari and non‐Qatari (Arab), males and females, age 18 years and above	Representative sample of Arabic speakers only: Qatari and non‐Qatari (Arab), males and females, age 18 years and above
Questionnaire	20 CIDI (version 3.3) and 5 non‐CIDI sections assessing the following: Sociodemographic, physical and mental health history, major depression, persistent depression, mania, generalized anxiety, panic, posttraumatic stress, social anxiety disorders, psychotic experiences, anger attacks, suicide, treatment, tobacco and drug use, employment, finance, personal relations, childhood trauma, Montreal Cognitive assessment, resilience and schizotypal personality questionnaire	16 CIDI (version 3.3) and 2 non‐CIDI sections assessing the following: Sociodemographic, physical and mental health history, COVID‐19 health, major depression, mania, generalized anxiety, panic, obsessive compulsive, posttraumatic disorders, psychotic experiences, treatment, employment, finance, personal relations, childhood trauma, and schizotypal personality questionnaire.
Study recruitment method	Support letter from the Ministry of public health along with a study brochure as part of a study information package given to participants on first household visit	SMS sent within 24 h ahead of first call and monetary incentives
Administration technology	The questionnaire was programmed using Blaise 5.2 (Blaise, 2017)—a multiplatform software. Computer Assisted personal interviewing (CAPI) for most survey instrument modules. Audio Computer‐Assisted Self‐Interviewing (ACASI) Blaise feature for a few sensitive modules	The questionnaire was programmed using Blaise 5.2 (Blaise, 2017)—a multiplatform software. All of the survey instrument modules were administered using Computer Assisted Telephone Interviewing (CATI)
Number of active survey interviewers	27 interviewers (20 female, 7 Male) 10 Head of groups	19 interviewers (17 females, 2 males)
Supervision structure	Two full‐time (unit manager and his assistant) managers, 4 supervisors (2 out of the 4 supervisors directly oversaw the male interviewers, while the remaining 2 oversaw the female interviewers and their head of groups)	Two full‐time (unit manager and his assistant) managers assisted by 3 student workers, 4 data quality monitors, and 6 direct supervisors. In addition, 3 research team members assisted the team by contributing more towards interview monitoring efforts
Data collection structure	Each interviewing group consisted of two female interviewers and one male head of group except for male interviewers who worked alone	Interviewers initially started from one sample but then the cases were programmatically bound to the interviewer of contact unless a gender match or, in some cases, dialect match was deemed appropriate
Quality control tool	The QCIS tool was developed for the CAPI mode in collaboration with IT teams at SESRI Qatar University and University of Michigan	The CAPI QCIS tool was adapted for CATI mode
Quality control indicators	Too many completed interviews per day Short question/stem question field High percentage of short field time Short/Long interview length Low prevalence rate/response rate Long pause	High number of completions Short question/stem question field time High percentage of short field time Short/Long interview length Low prevalence rate Long question field/treatment length time Multiple field/stem field visits

Abbreviations: CAPI, Computer‐Assisted Personal Interviewing; CATI, Computer‐Assisted Telephone Interviewing; CIDI, Composite International Diagnostic Instrument; QCIS, Quality Control Interviewing System.

A survey's sample design, including the sampling strategy, was also influenced by the mode, as the information used to improve sampling efficiency differs between modes. As mentioned earlier, a detailed description of the sample design is described elsewhere (Khaled et al., 2024).

### 2.2 Survey questionnaire

We adapted the WMHQ survey instrument to phone mode through three main modifications. First, we revised the survey introduction. In phone surveys, the initial interaction with the interviewer typically starts with a skilled solicitation, but then shifts to a neutral and professional tone. As a result, our team modified the study questionnaire's introductory section to increase survey salience and align with the requirements of phone interviews. Second, we aimed to reduce the overall interview duration by an average of approximately 30 min compared to the previously piloted face‐to‐face survey instrument. Third, we wanted to incorporate COVID‐19‐related content to address the psychological impact of the crisis on respondents' mental health and capture any other pandemic‐associated symptoms.

The final questionnaire was reduced from 25 (face‐to‐face pilot) to 18 (phone pilot) total modules. This reduction involved the removal of nine (CIDI and non‐CIDI) modules, including suicide, persistent depression, anger attacks, social anxiety, and tobacco and drug use. In their place, we added two new modules, focusing on assessing the psychological toll of COVID‐19 and diagnostic criteria for obsessive‐compulsive disorder.

### 2.3 Recruitment methods

Even after shortening the WMH questionnaire for phone mode, it remained quite long, taking 50–60 min to complete, in contrast to the typical 20–25 min duration of a standard phone survey. Consequently, the telephone pilot included an experiment that employed a gift‐based incentive to boost the participation rate. The incentive offered was an electronic gift card for food outlets (one card per eligible participant) valued at 14 US dollars (USD), delivered via SMS text, or the option to donate the equivalent value of the gift card to a Qatar‐based charity of their choice.

Compared to the face‐to‐face pilot, technology played a larger role in the recruitment process for the phone pilot. We initiated the process by sending a Short Message Service (SMS) text to each eligible respondent 24 h prior to the first interview phone call. We chose to use an SMS because the length and sensitivity of the questions made it essential to increase the salience of the study by leveraging the prominence of the survey's sponsors, which we believed would reassure respondents prior to our call.

The SMS served a dual purpose: It not only informed potential respondents of the upcoming study participation call, but also provided a link to the study website, which helped explain the purpose of the survey. However, some eligible participants expressed security concerns regarding the link. Many hesitated to click it until they had been contacted by an interviewer and received reassurance about the link's authenticity. Individual SMS requests were also sent daily to any respondents who wanted to participate but were not able to find the original text message or had concerns about its source.

### 2.4 Fielding team

Due to the pandemic, the phone survey lab at Qatar University's Social and Economic Survey Research Institute (SESRI) had to be temporarily closed. Consequently, SESRI initiated and tested a direct dialing phase for a distributed (remote) Computer Assisted Telephone Interviewing (CATI) system during the summer of 2020. This was done in preparation for the phone pilot scheduled for the fall of 2020.

During the phone pilot, SESRI interviewers were able to make calls to respondents safely from their homes rather than from the phone lab. They were closely monitored by field supervisors using this remote capability. This approach allowed the study team to collect data over the phone while adhering to pandemic‐related social distancing policies.

As indicated in Table 1, the size of the fielding team is smaller in the phone pilot compared to the face‐to‐face pilot. However, the interviewer‐to‐supervisor ratio was higher for the phone pilot. This structural adjustment was necessary to facilitate more extensive remote verification activities. These activities included ensuring interviewers adhered to their working schedules, met the required minimum number of working hours, and were subjected to live monitoring of calls by supervisors.

The phone pilot involved an unusually long phone survey on a sensitive topic. Accordingly, the team placed increased emphasis on monitoring how questions were asked, the pace of the interviews, and identifying any irregularities in the collected data as crucial aspects of quality control. Therefore, we increased the proportion of live call monitoring sessions by involving research team members to assist the supervisors in this activity.

### 2.5 Response rate & field cost

The response rates (RR) were calculated using standardized coding and interpretation procedures for different calling outcomes, following the guidelines set by the American Association for Public Opinion Research (AAPOR, 2015). Completed responses included those who finished the whole survey questionnaire (reaching the last question in the survey). Those who did not complete the survey interview were divided into three categories: eligible, ineligible, and cases of unknown eligibility. Eligible respondents (“eligibles”) included Arab residents who either refused to participate in the study, agreed to an appointment, but did not fulfill it upon follow‐up, or completed part of the interview. Ineligible respondents (“ineligibles”) included mostly non‐Arabs and those under 18 years of age. Unknown eligibility cases (“unknowns”) encompassed housing units with no one at home (in the face‐to‐face survey) or phone numbers with no answer (in the phone survey). Those who immediately refused to participate in the survey before interviewers were able to identify their eligibility were also included in this category.

We report two response rates in Table 3. First, the raw response rate, which is the ratio between the number of completions and total sample sizes after excluding ineligibles: RR1 = C/(C + E + UE) where C is the number of completions, E is the number of eligible responses, and UE is the number of unknown eligibility. Second, the adjusted response rate, which is RR2 = C/(C + E + eUE) where e is the estimated proportion of eligibilities given by e = (C + E)/(C + E + IE) where IE is the number of ineligible cases.

The break‐off rate is calculated by dividing the number of break‐offs by the sum of the number of break‐offs and the number of completions. The break‐off group includes people who agreed to participate in the survey, answered some questions, but did not complete the entire survey interview.

The cost per completion was calculated using the total survey fielding cost divided by the number of completions. The field costs only cover payments made to supervisors and interviewers for their working hours during training and fielding. The cost per completion does not include any costs associated with questionnaire development, sampling, training, programming, or administrative activities. We focus on the field cost to compare the two pilots because, as the study transitions from the pilot to full‐scale production, only the field cost will grow rapidly while other costs remain relatively stable.

### 2.6 Physical and mental health problems

During the study, two different sources were used to identify the history of physical and mental health disorders. The first source included direct responses from the respondents about whether they have ever been diagnosed by a health professional with major depression, panic attacks, post‐traumatic stress disorder, obsessive‐compulsive disorder, generalized anxiety disorder, mania, bipolar disorder, schizophrenia, or any other emotional problems. To ascertain the history of having any physical chronic condition, respondents were asked if they had any life‐threatening or seriously impairing chronic physical health problems such as cancer, heart disease, or lung disease. Then respondents were able to choose from a list of physical diseases the type of physical illness they had at the time of the interview (if any).

The second source was responses to the diagnostic modules within the interview, which were used to calculate the prevalence of experiencing any mental disorder by the time of the interview. These were based on the DSM‐5 criteria, as outlined in CIDI (version 3.3). Based on the modules assessed in both pilots, we defined three main groups of mental disorders. Any anxiety disorder included meeting diagnostic criteria for any of the following conditions: generalized anxiety disorder, panic disorder, and post‐traumatic stress disorder. Any mood disorder included meeting diagnostic criteria for any of the following conditions: major depressive disorder, bipolar I‐II disorders. Any disorder was defined as meeting diagnostic criteria for any of the abovementioned anxiety and/or mood disorders.

As we aimed to examine survey mode effects, we needed to account for the effect of COVID‐19 on the prevalence estimates in the phone pilot survey conducted during the pandemic. For this purpose, we identified and excluded 70 cases (16%) who reported the onset of mood and anxiety disorders during the pandemic period only. Since all the interviews were conducted during the COVID‐19 pandemic period (2019–2022), we estimated the lifetime prevalence of the assessed disorders while excluding cases whose age of onset for any disorder occurred only during the pandemic period. Therefore, only cases that met CIDI criteria for any disorder up to 2 years preceding the interview date were included and counted toward the lifetime prevalence rate.

### 2.7 Sociodemographic variables

In both pilots, we assessed the same basic sociodemographic variables, including age, gender, marital status, education, employment, income, and nationality. Qatar's income categories were constructed in reference to Qatar's census income data. For example, more than half of the categories of income are less than the median personal earnings of 20,000 Qatari Riyals (QAR), equivalent to 5400 US dollars (USD). Similarly, other variables were adapted and modified for Qatar's context. For example, employment questions were adapted to reflect job categories and working hours in accordance with Qatar's employment system. Additionally, response options for the marital status question were slightly modified to reflect sanctioned cultural and religious aspects of marriage within the context of Qatar.

### 2.8 Statistical analysis

We report descriptive statistics, including frequencies, percentages, and corresponding 95% confidence intervals (CI). All estimates were weighted to account for the sampling design in each pilot. We compared proportions using *p*‐values based on the F‐transformed version of the Pearson Chi‐square statistic, with a significance level defined at 0.05 for a two‐tailed test. All statistical analyses were performed using Stata Software version 16 (Stata, 2016).

## 3 RESULTS

Table 2 shows the distribution of both pilot samples across basic sociodemographic variables. Those who were 18 to 44 years of age constituted 74.9% of the total sample in the face‐to‐face pilot, compared to 68.1% in the phone pilot. Estimates for face‐to‐face versus phone were similar on the following variables: single marital status (20.8% vs. 19.1%), secondary level of education or lower (38.7% vs. 35.6%), unemployed (37.2% vs. 32.3%), monthly income less than 20,000 Qatari Riyals (33.5% vs. 36.5%), and Qatari nationality (29.0% vs. 26.4%). Females represented 48.6% of the total sample in the face‐to‐face pilot, compared to 40.0% in the phone pilot. With the exception of the gender comparison (*p* = 0.040), none of the other comparisons were statistically significant.

**TABLE 2:** Sociodemographic characteristics of Qatar's national mental health survey pilots.

		Face‐to‐face pilot			Telephone pilot			*p*‐value
Sociodemographic characteristics		Freq.	%	95% CI	Freq.	%	95% CI	
Age group (years)	18‐44	259	74.9	(69.0–80.1)	299	68.1	63.2–72.6)	0.070
	45+	90	25.1	(19.9–31.0)	128	31.9	(27.4–36.8)	
Gender	Female	187	48.6	(42.0–55.2)	167	40.0	(35.2–45.0)	0.040
	Male	162	51.4	(44.8–58.0)	260	60.0	(55.0–64.8)	
Marital status	Single	44	20.8	(15.0–28.3)	84	19.1	(15.6–23.3)	0.656
	Ever Married	304	79.2	(71.7–85.0)	343	80.9	(76.7–84.4)	
Education	Secondary or less	118	38.7	(32.3–45.6)	151	35.6	(31.0–40.5)	0.454
	Diploma+	228	61.3	(54.4–67.7)	276	64.4	(59.5–69.0)	
Employment	Employed	212	62.8	(56.4–68.9)	288	67.7	(62.8–72.2)	0.226
	Unemployed	134	37.2	(31.1–43.6)	135	32.3	(27.8–37.2)	
Monthly income (Qatari riyals/USD $)	Less than 20k QAR (≤5400 USD)	96	33.5	(26.7–41.1)	101	36.5	(31.0–42.2)	0.524
	More than 20k QAR (>5400 USD)	238	66.5	(58.9–73.3)	208	63.5	(57.6–69.0)	
Nationality	Qatari citizen	63	29.0	(22.2–37.0)	95	26.4	(22.1–31.3)	0.549
	Arab nationality	286	71.0	(63.0–77.8)	332	73.6	(68.7–77.9)	

Abbreviations: CI, Confidence Interval; QAR, Qatari Riyals; %, Percentage.

As shown in Table 3, the raw response rates for the face‐to‐face and phone pilots were 32.4% and 18.8%, respectively. While the feasibility of conducting a lengthy phone interview was established, the incentive was deemed ineffective in increasing the participation rate and thus was not used in the actual survey production.

**TABLE 3:** Survey measures descriptive statistics by survey mode.

Survey measures	Survey mode	
Disposition	CAPI	CATI
Complete	349	426
Not complete		
Eligible	99	927
Ineligible	202	1884
Unknown	426	913
Total	1076	4150
Response		
Raw response rate (%)	32.4	18.8
Adjusted response rate (%)	47.1	24.6
Break‐off rate (%)	4.6	49.2
Other		
Field duration (Days)	15	24
Maximum number of attempts	3	7
Median interview length (minutes)	92	80
Fielding cost measures		
Number of interviewers and supervisors	43	29
Number of work hours	3784	2883
Field cost (US dollars)	57,544	31,997
Number of completions	349	426
Field cost per completion (US dollars)	164.9	75.1

The response rate was almost two times higher for the face‐to‐face pilot compared to the phone pilot, with the following adjusted response rates: 47.1% and 24.6%, respectively (Table 3). The difference in response rates between the two modes can be explained by the break‐off rate difference. In the face‐to‐face pilot, this rate was only 4.6%, while the same number for the phone pilot was much higher at 49.2%. In our calculations, if the break‐off rate in the phone pilot was the same as the face‐to‐face pilot, then the response rate could be similar between the two pilots.

We compared the fielding costs between the two pilots. The main cost indicator in Table 3 is the field cost per completion in the last row. The face‐to‐face pilot's cost was more than double that of the phone pilot, at 164.9 USD versus 75.1 USD, respectively. This suggests that the field cost during full‐scale production would be much larger for the face‐to‐face survey. Such a substantial cost difference could render a face‐to‐face survey financially infeasible, while a phone survey may remain a viable option.

The duration for fielding the surveys was 24 days for the phone pilot and 15 days for the face‐to‐face pilot (Table 3). The average duration of the phone interview was approximately 77 min, compared to 97 min for the face‐to‐face interview. The maximum number of contact attempts was three in the face‐to‐face pilot compared to seven in the phone pilot (Table 3).

As shown in Table 4, the prevalence of any lifetime mental disorder, specifically mood or anxiety disorders, reported by the participant as diagnosed by a health professional was 11.3% in the face‐to‐face pilot compared to 12.0% in the phone pilot (*p* = 0.793). The percentage of respondents who reported having one mental disorder only diagnosed by a health profession was 6.6% in both the face‐to‐face and phone pilots. Meanwhile, the percentages for two or more reported disorders were 4.7% in the face‐to‐face pilot compared to 5.4% in the phone pilot (*p* = 0.921), respectively.

**TABLE 4:** Survey mode comparisons for any lifetime and number of mood, anxiety, or physical disorders as diagnosed by a health professional.

		Survey mode						*p*‐value
		Face‐to‐face (*n* = 349)			Telephone (*n* = 396)			
		Freq.	%	95% CI	Freq.	%	95% CI	
Any mood or anxiety disorder	Yes	44	11.3	8.1–15.5	45	12.0	8.9–15.9	0.793^a^
	No	305	88.7	84.5–91.8	351	87.9	84.0–91.1	
Number of mood or anxiety disorders	0	305	88.7	84.5–91.8	351	88.0	84.0–91.1	0.921^c^
	1	27	6.6	4.3–10.0	25	6.6	4.3–9.9	
	2+	17	4.7	2.7–7.9	20	5.4	3.4–8.4	
Any life‐threatening or seriously impairing chronic physical health condition	Yes	31	7.8	5.3–11.3	42	10.4	7.8–14.1	0.238^b^
	No	318	92.2	88.7–94.7	354	89.6	85.9–92.2	
Number of life‐threatening or seriously impairing chronic physical health conditions	0	319	92.4	88.8–94.8	354	89.6	86.0–92.3	0.436^d^
	1	21	5.3	3.3–8.4	31	7.6	5.3–10.8	
	2+	9	2.3	1.1–4.9	11	2.8	1.5–5.2	

^a^ Uncorrected *χ* ^2^ = 0.084, Degrees of freedom = 1, Design‐corrected *F* (1, 744) = 0.069.

^b^ Uncorrected *χ* ^2^ = 1.562, Degrees of freedom = 1, Design‐corrected *F* (1, 744) = 1.396.

^c^ Uncorrected *χ* ^2^ = 0.199, Degrees of freedom = 2, Design‐corrected *F* (2, 1487) = 0.083.

^d^ Uncorrected *χ* ^*2*^ = 1.882, Degrees of freedom = 2, Design‐corrected *F* (2, 1485) = 0.830.

Table 4 also shows that the prevalence of any chronic physical condition, reported by the participant as diagnosed by a health professional, was 7.8% in the face‐to‐face pilot compared to 10.4% in the phone pilot (*p* = 0.238). In the face‐to‐face pilot, 5.3% of respondents reported having only one chronic physical condition diagnosed by a health professional, compared to 7.6% for the phone. For those reporting two or more conditions, the percentages were 2.3% in the face‐to‐face compared to 2.8% in the phone pilot (*p* = 0.436).

As shown in Table 5, the prevalence of any mood or anxiety disorder as defined by the CIDI were also similar in the face‐to‐face (19.3%) and phone (22.7%) pilots (*p* = 0.305). Also shown in Table 5, the number of disorders as per CIDI criteria were also similar across the two modes with 4.9% of the sample in the face‐to‐face pilot meeting criteria for two or more disorders compared to 5.5% in the phone pilot (*p* = 0.579). Furthermore, similar results were obtained when stratifying the results by gender as shown in Appendix Table S1 (Male) and Appendix Table S2 (Female).

**TABLE 5:** Survey mode comparisons for any lifetime and number of mood or anxiety disorders as per composite international diagnostic instrument (CIDI) criteria.

		Survey mode						*p*‐value
		Face‐to‐face (*n* = 349)			Telephone (*n* = 396)			
		Freq.	%	95% CI	Freq.	%	95% CI	
Any mood or anxiety disorder	Yes	76	19.3	15.1–24.4	88	22.7	18.6–27.4	0.305^a^
	No	273	80.7	75.6–84.9	308	77.3	72.6–81.4	
Number of mood or anxiety disorders	0	273	80.7	75.6–84.9	308	77.3	72.6–81.4	0.579^b^
	1	59	14.4	10.9–18.8	67	17.2	13.6–21.6	
	2+	17	4.9	2.9–8.3	21	5.5	3.5–8.4	

*Note*: All the percentages were weighted to account for sampling design in both surveys. Mood or anxiety disorder was defined based on meeting DSM‐5 criteria as measured by the composite international diagnostic instrument (version 3.3) for any of the following disorders: major depressive disorder, bipolar I/bipolar II, generalized anxiety disorder, panic disorder, and post‐traumatic disorder.

^a^ Uncorrected *χ* ^2^ = 1.262, degrees of freedom = 1, Design‐corrected *F* (1, 744) = 1.052.

^b^ Uncorrected *χ* ^2^ = 1.305, degrees of freedom = 2, Design‐corrected *F* (2, 1486) = 0.547.

## 4 DISCUSSION

This study investigated the feasibility of substituting telephone interviews for face‐to‐face interviews in the WMHQ survey during the COVID‐19 pandemic. We assessed the net effect of survey mode by addressing the practical question of whether the resulting prevalence estimates of the two main classes of mental disorders in the WMHQ (mood and anxiety disorders) are similar or different across the two pilots. This assessment was made irrespective of pandemic‐related influences on the prevalence of mental disorders or any specific methodological reasons behind these differences. We also compared response rates and fielding costs across modes.

The main source of variance in the methodological aspects of both pilots stemmed from differences in the survey modes of the two pilots, including factors such as questionnaire length, study recruitment method, and fielding team size and structure. These aspects affected the survey response rate and field costs of both pilot studies.

While the face‐to‐face pilot generated a response rate two to three times higher than the phone pilot, the total fielding (variable) costs in the face‐to‐face mode were just over two times that of the phone. Lower response rates in telephone surveys compared to face‐to‐face are consistent with previous studies (Groves & Kahn, 1979; Hox & De Leeuw, 1994a). The difference in response rate is largely attributed to the more personal nature of face‐to‐face interviews relative to phone (Drolet & Morris, 2000). Notably, the response rates between the two modes in our study were initially similar, but then diverged after accounting for the much higher break‐off rate for the phone compared to face‐to‐face. In the face‐to‐face pilot, social norms likely made it less acceptable for participants to discontinue the interview after they had invited interviewers into their homes. Conversely, in the phone survey, participants found it much easier to terminate the interview at any point. Given the survey's lengthy and sensitive questionnaire, break‐offs were more likely, especially for the phone pilot.

The higher risk of break‐offs for telephone surveys relative to in‐person interviewing is well documented in survey research. It is easier to end a phone interview by simply hanging up, and the act of talking on the telephone for extended periods can be especially tiring for some respondents (Holbrook et al., 2003). For the WMHQ survey, the median interview length was 80 min for the phone pilot and 90 min for the face‐to‐face pilot. The eligibility of potential respondents is also less frequently known for a phone survey compared to face‐to‐face. A non‐contact in a phone survey generally provides little or no information about eligibility compared to face‐to‐face, where the eligibility of the household can often be determined by interviewers through observation of the characteristics of the property. This was in fact the case for our study; the percentage of unknown eligibility in our phone sample was much higher than in the face‐to‐face sample.

Our results are consistent with the generally recognized higher costs of conducting face‐to‐face interviews relative to phone. Furthermore, the ratio of costs is also on par with what has been reported in the literature of around 2 to 1 (van Campen, 1998; Warner et al., 1983; Weeks et al., 1983). This study, to our knowledge, is the first published to date that compares fielding costs per completion by mode within the WMH survey consortium. Arguably, the cost savings of conducting a lengthy survey over the phone can be reinvested into enhancing interviewer supervision, study visibility, and respondent assistance.

This reinvestment approach was employed in our phone study, where we allocated more resources to quality control monitoring, including a higher proportion of live interview monitoring and the use of technologies to capture and assess in real‐time quality indicators from paradata and survey data. These indicators, in turn, highlighted which interviewers needed more attention to correct undesirable and potentially bias‐inducing behavior. The advertisement budget for the study was also robust, as was the investment in handling respondent questions and concerns. It is worth noting, however, that costs for the telephone‐based survey increased further over time during the production phase, as detailed in another manuscript in this journal issue (Khaled et al., 2024).

Regarding differences in demographic variable distributions across the two modes, the two samples were similar on most basic sociodemographic characteristics, except for gender. We found a statistically significant gender difference, with males constituting a higher percentage of the phone respondents than in the face‐to‐face sample (60% vs. 51%). This finding is also consistent with previous literature showing that males are somewhat more likely to participate in phone surveys compared to face‐to‐face surveys (Aneshensel et al., 1982; Ellis & Krosnick, 1999; Groves & Kahn, 1979; Weeks et al., 1983).

In terms of the Middle East and Qatar in particular, men are generally less inclined to participate in research and probably even less likely to participate in mental health research because of negative cultural attitudes and stigma against mental illness (Zolezzi et al., 2017). Therefore, participation gains among members of this group of the target population are advantageous. The increased privacy offered by the phone may lead to higher participation among males than in face‐to‐face surveys and perhaps even more accurate reporting of less socially desirable attributes related to the symptoms and burden of mental illness. However, there is no way of ascertaining the latter possibility from our study.

Finally, in addressing the crucial question of whether mode effects would influence the main survey estimates of interest for the study, our findings are largely reassuring. Both modes resulted in similar lifetime prevalence estimates of mental illness for the two main classes of disorders assessed in the WMHQ. Surveys conducted within the WMH consortium are known for their high quality and rigor in terms of estimates of mental illness prevalence and their associations with risk factors for mental illness (Kessler et al., 2009). However, to date, none of these surveys have used the telephone as the primary data collection mode. It is standard practice in the WMH surveys that, while the majority of the interview is completed face‐to‐face, long interviews requiring multiple visits to a household may be completed using a telephone follow‐up. These results also logically support the validity of using the phone in this secondary role as well.

Importantly, in our study, the distribution of respondents across the number of disorders, which relates to severity of symptoms and burden of illness, were the same across both modes. This finding reassures us of the similar overall quality of the responses and supports the viability of a phone survey as a credible alternative to face‐to‐face. This is especially apparent during a pandemic like COVID‐19 or any other condition where costs or practical considerations limit in‐person efforts.

## 5 CONCLUSIONS

Born of necessity, two large probability‐based pilots were conducted prior to and during the COVID‐19 pandemic, using different modes of data collection. These allowed us to compare and contrast the methodological aspects of each mode. To our knowledge, this study is the first to provide evidence supporting the feasibility of telephone interviewing as a substitute for face‐to‐face interviews within the WMH survey initiative. The study's findings confirm that telephone interviews can yield similar criterion‐based mental disorder prevalence estimates as face‐to‐face interviews. There are some caveats and limitations, however.

First, this survey targeted an Arabic‐speaking population in a region with relatively high telephone response rates compared to many Western countries. Second, it was conducted under conditions permitting a high‐coverage, relatively efficient cellular phone sample. Third, the savings from not conducting a more costly face‐to‐face survey were in part reallocated to robust quality monitoring, advertising, and respondent outreach. This is necessary to offset the difficulties of gaining and sustaining cooperation for a long interview on a sensitive topic. In addition to these costs, pandemic conditions require a distributed network CATI system so that interviewers and their supervisors can work remotely. Such a system tends to be more costly to administer than a centralized calling lab.

A theoretical limitation is that there was no experimental assignment of mode. The two samples are drawn using different methods, and even if this issue could be overcome through some other sampling method, such as address‐based (ABS), this comparison arose due to the unexpected and sudden onset of a pandemic. The latter precluded a fully experimental design. With those caveats, overall, this study lends support to the feasibility of adopting a phone strategy for future mental health surveys where a probability sample is desired in the context of future pandemics. It also informs of the potential for changing the way data are collected under conditions of necessity, even for very long and sensitive studies like those typically administered within the WMH consortium.

## AUTHOR CONTRIBUTIONS

**Salma M. Khaled**: Conceptualization; funding acquisition; writing ‐ original draft; methodology; writing – review & editing; project administration; supervision; data curation; resources. **Iman Amro**: Writing – original draft; writing – review & editing; methodology; project administration. **Lina Bader**: Writing – review & editing; formal analysis; software. **John Lee Holmes**: Writing – original draft; writing – review & editing; project administration; data curation. **Abdoulaye Diop**: Writing – review & editing; project administration; resources. **Kien Le Trung**: Writing – original draft; writing – review & editing; project administration; conceptualization.

## CONFLICT OF INTEREST STATEMENT

None.

## ETHICS STATEMENT

Qatar University (QU‐IRB 1219‐EA/20) approved the study. The study's goal and methods were verbally explained to participants. Before each survey interview, consent to participate was verbally obtained using a phone script. All data were encrypted and saved on Qatar University's secure server. Each participant was assigned a case number, and individual identifiers were retained in a password‐protected folder only available to the lead principle investigator, senior research assistant, and data analyst. All study researchers, including interviewers, signed confidentiality agreements preventing the sharing or use of participant personal information.

## Supporting information

Supporting Information S1

## Data Availability

The data that support the findings of this study are available from Dr. Salma M. Khaled, the principal investigator of the study, at skhaled@qu.edu.qa, upon reasonable request and pending additional ethical approval.
