# Supplementary material for: Feasibility of replacing face‐to‐face with telephone interviews for the World Mental Health Qatar survey during the COVID‐19 pandemic
Source: Int J Methods Psychiatr Res. 2024 May 10;33(Suppl 1):e2009. doi: 10.1002/mpr.2009 (PMC11323766; doi:10.1002/mpr.2009)
Supplement: Supplementary file 1 — Supporting Information S1 [file MPR-33-e2009-s001.docx]

**Appendix Table 1.** Survey Mode Comparisons for Any Lifetime and Number of Mood or Anxiety Disorders as per Composite International Diagnostic Instrument (CIDI) Criteria (Male)

|  | | ***Survey Mode*** | | | | | | ***p-value*** | |
| --- | --- | --- | --- | --- | --- | --- | --- | --- | --- |
|  |  | ***Face-to-Face (n=162)*** | | | ***Telephone (n=245)*** | | |  |  |
|  |  | ***Freq.*** | ***%*** | ***95% CI*** | ***Freq.*** | ***%*** | ***95% CI*** |  |  |
| ***Any mood or anxiety disorder*** | ***Yes*** | *25* | *16.0* | *7.7 -18.4* | *43* | *17.8* | *13.3 - 23.4* | 0.134^a^ |  |
|  | ***No*** | *137* | *88.0* | *81.6 - 92.3* | *202* | *82.2* | *76.6 - 86.7* |  |  |
| ***Number of Mood or Anxiety Disorders*** | ***0*** | *137* | *88.0* | *81.6 - 92.3* | *202* | *82.2* | *76.6 - 86.7* | 0.231^b^ |  |
|  | ***1*** | *19* | *8.3* | *5.0 - 13.6* | *34* | *14.1* | *10.1 - 19.3* |  |  |
|  | ***2+*** | *6* | *3.7* | *1.5 - 8.7* | *9* | *3.7* | *1.9 - 7.1* |  |  |
| ***Note****. All the percentages were weighted to account for sampling design in both surveys.*  *Mood or anxiety disorder was defined based on meeting DSM-5 criteria as measured by the composite international diagnostic instrument version 3.3 for any of the following disorders: major depressive disorder, bipolar I /bipolar II, generalized anxiety disorder, panic disorder, and post-traumatic disorder* | | | | | | | | | |
| ^a^Uncorrected Chi ^2^ = 2.629, Degrees of freedom = 1, Design-corrected F(1, 406) = 2.251  ^b^Uncorrected Chi^2^ = 3.363, Degrees of freedom = 2, Design-corrected F(2, 809.6) = 1.468 | | | | | | | | | |

**Appendix Table 2.** Survey Mode Comparisons for Any Lifetime and Number of Mood or Anxiety Disorders as per Composite International Diagnostic Instrument (CIDI) Criteria (Female)

|  | | ***Survey Mode*** | | | | | | ***p-value*** |
| --- | --- | --- | --- | --- | --- | --- | --- | --- |
|  |  | ***Face-to-Face (n=187)*** | | | ***Telephone (n=151)*** | | |  |
|  |  | ***Freq.*** | ***%*** | ***95% CI*** | ***Freq.*** | ***%*** | ***95% CI*** |  |
| ***Any mood or anxiety disorder*** | ***Yes*** | *51* | *27.0* | *20.4 - 34.8* | *45* | *30.2* | *22.9 - 38.6* | 0.568^a^ |
|  | ***No*** | *136* | *73.0* | *65.2 - 79.6* | *106* | *69.8* | *61.4 - 77.1* |  |
| ***Number of Mood or Anxiety Disorders*** | ***0*** | *136* | *73.0* | *65.2 - 79.6* | *106* | *69.8* | *61.4 - 77.1* | 0. 782^b^ |
|  | ***1*** | *40* | *20.8* | *15.0 - 28.0* | *33* | *14.1* | *15.6 - 30.0* |  |
|  | ***2+*** | *11* | *6.2* | *3.2 - 11.8* | *12* | *3.7* | *4.6 - 14.2* |  |
| ***Note****. All the percentages were weighted to account for sampling design in both surveys.*  *Mood or anxiety disorder was defined based on meeting DSM-5 criteria as measured by the composite international diagnostic instrument version 3.3 for any of the following disorders: major depressive disorder, bipolar I /bipolar II, generalized anxiety disorder, panic disorder, and post-traumatic disorder* | | | | | | | | |
| ^a^Uncorrected Chi^2^ = 0.394, Degrees of freedom = 1, Design-corrected F(1, 337) = 0.327  ^b^Uncorrected Chi^2^ = 0.601, Degrees of freedom = 2, Design-corrected F(2, 673.8) = 0.246 | | | | | | | | |
